# Perimesencephalic Hemorrhagic-Pontine Infarction Syndrome: Miler Fisher's Hint to Subarachnoid Paramedian Artery Rupture—A Case Report

**DOI:** 10.1155/crnm/3384633

**Published:** 2025-11-14

**Authors:** R. Targa Martins, C. Oliveira

**Affiliations:** Neurology and Stroke Unit Department, Hospital Nossa Senhora da Conceição, Grupo Hospitalar Conceicao, Porto Alegre, Rio Grande Do Sul, Brazil

**Keywords:** case report, paramedian pontine infarction, perimesencephalic subarachnoid hemorrhagic

## Abstract

We report a 73-year-old man transferred for evaluation of suspected aneurysmal subarachnoid hemorrhage after his first-ever thunderclap headache episode. It was noted on the second day of the disease left hemiparesis. On MRI, the hemorrhage was of the perimesencephalic type and was associated with an early right paramedian pontine infarction. Angiography did not reveal an aneurismal source for the bleeding, arterial dissection, nor vertebrobasilar vasospasm. Synchronic pontine infarction with perimesencephalic hemorrhage is an unusual syndrome ascribed to the rupture of a perforator superficial arterial segment, as described by Hochberg and Miller Fisher in a case report with autopsy. The absence of a bleeding source for subarachnoid hemorrhage and the presence of ischemic paramedian pontine perforator reinforce the role of artery rupture in the etiology of this case in particular but also as the main cause of concomitant hemorrhagic-ischemic brainstem syndrome. The patient had a satisfactory recovery and was treated with antiplatelet therapy, statins, and rehabilitation. Paramedian pontine infarction and perimesencephalic hemorrhage should be considered a concomitant hemorrhagic-ischemic syndrome suggesting basilar perforator rupture as the etiologic mechanism of the stroke, although rare.

## 1. Introduction

Perimesencephalic subarachnoid hemorrhage (PSAH) comprises about 20% of nonaneurysmal SAH [1]. PSAH etiology includes venous drainage abnormality, basilar artery ectasia, and basilar perforator superficial rami rupture (PSR) in its short subarachnoid portion. We describe PSAH presenting with concomitant paramedian pontine infarction (PMI) and typical perforator basilar rami pathology as described by Hochberg and Miller Fisher in 1974.

We report a 73-year-old right-handed man transferred to a tertiary center to evaluate SAH etiology with a sudden ‘Thunderclap-'type headache more intense in the neck and occiput and confusion 3 days before admission to our hospital after first evaluation in his local facility. He presented with neck rigidity, severe hypertension (220/140), agitation, a Glasgow Coma Scale of 13 (eye 3, verbal 4, and motor 6), and left-sided paresis (NIHSS 5: orientation 1, reduced horizontal gaze to the right 1—possible right abducens paresis, left arm: 1 point, left leg: 1 point, and face left: 1 point), and started with intravenous anti-hypertensive therapy and phenytoin. He suffered from hypertension and prostate hyperplasia without known vascular complications, and he was treated irregularly with losartan plus hydrochlorothiazide and doxazosin. He did not use anticoagulation therapy or suffer from polycystic kidney disease. He never had a stroke, systemic bleeding, or acknowledged relatives with SAH or intracranial aneurysm. Head computed tomography (CT) at admission showed pretruncal (mesencephalon, pons, and medulla) SAH extending to the Sylvian fissure, distal ectatic basilar artery with wall atheroma. On the third day after symptom onset, he was transferred to our tertiary neuro-ICU to evaluate the possible top-of-basilar aneurysmatic SAH. He presented with a lowered level of consciousness (GCS: 9), unchanged left hemiparesis, isochoric and reactive pupils, a Fisher Scale of IV, and a Hunt Hess Scale of 3. He underwent endotracheal intubation and was submitted to brain CT, CTA, and his first four-vessel angiography.

**Figure 1:**
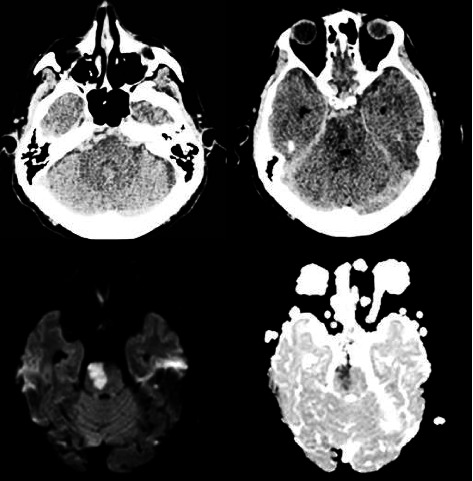
Admission brain CT-Day 3 and MRI Day 4 after symptom onset showing pretruncal perimesencephalic subarachnoid hemorrhage and paramedian pontine infarction.

**Figure 2:**
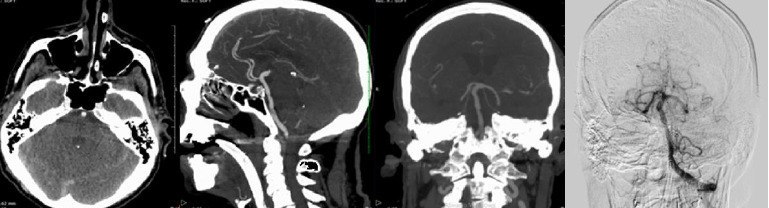
Right panel acute CT-angio (Day 3) and digital angiography (Day 4) showing the atherosclerotic vertebrobasilar system without bleeding source, dissection, or early vasospastic changes.

Early CT and MRI revealed, besides PSAH without a bleeding source, a right acute PMI congruent with right abducens ophthalmoparesis and left motor syndrome presentation. There were deep hemispheric white matter hyperintensities, enlarged perivascular spaces, and lacunes characteristic of small vessel disease. The main angiography findings were the absence of an aneurysmal source of SAH bleeding or detectable vasospasm, specifically in posterior circulation where the blood was present. Visible basilar irregularities were interpreted as atherosclerotic disease. Twenty-two days after the onset of symptoms, angiography was repeated without any modification, reinforcing the impression of basilar wall atheroma rupture without vasospasm, vessel wall dissection, or mural hematoma (Figures 1 and 2).

The patient was started on nimodipine, antiplatelet therapy with acetylsalicylic acid, and needed treatment to nosocomial pulmonary infection. He gradually improved his neurologic status and was discharged home on the 24th day with minor left hemiparesis, oriented and a modified Rankin Scale of 2, with antiplatelet, antihypertensive, and statin treatment.

## 2. Discussion

Our report describes easily recognized hemorrhagic and ischemic syndromes: PMSAH and PMI presenting synchronously. This association was described in a pathological report but is uncommonly reported [2–5], summing up to 5 cases so far and deserves attention for faster identification of small vessel disease as a common etiology for earlier therapeutic measures not focusing only on ischemic or hemorrhagic aspects of presentation. In an earlier description of the “meningeal type” of cerebral hemorrhage in 1929, Bagley first postulated that “direct rupture of an atherosclerotic vessel” was a cause of one SAH in the carotid circulation [2]; however, only in 1974 did Hochberg and C.M. Fisher report a hemorrhagic-ischemic presentation with a pathological detailed description of a 150-μm diameter short circumferential artery rupture in its superficial subarachnoid portion [3]. Since then, Tatter in 1995 emphasized the pathophysiologic aspects of undefined source SAH and the PSR role when concomitant ischemia is present soon after bleeding in chronic hypertensive arteriopathy. His two cases were mainly of anterior circulation ischemia at onset but one case developed medullary infarction after a late angiography [4]. Only in 2007, Lansberg describe the second typical paramedian pontine perimesencephalic hemorrhagic ischemic case reinforcing the most plausible etiology as artery rupture instead of focal dissection [5]. Alexander et al. reviewed 140 cases of negative pan-angiography SAH and pointed to perforator arteries (ventriculostriate and thalamoperforating groups) as potential leaking sources [6]. Mohan et al., in a recent review of nonaneurysmal PMSAH, estimated the incidence of vasospasm and ischemic complications to be 9% [7]. Another review by Hou and Yu notes the absence of early vasospasm with this complication usually beginning on the fourth day after bleeding and peaking on the seventh, and early focal signs correlate mainly with acute cranial nerve palsies [8]. Other possible etiologies of PMSAH described in the neuroradiological literature—abnormal venous drainage or mural basilar hematoma draining to the subarachnoid space—are not associated with or systematically related to acute ischemic events. Castillo recently reported a concomitant sulcal SAH and hemispheric ischemic stroke case in a severe COVID-19 case and attributed it to possible inflammatory vasculopathy—coagulopathy [9]. We did not find any suggestion of dissection in 2 cerebral angiograms 22 days apart nor in acute brain MRI adding strength to the construct of artery rupture as the cause of the clinical presentation. Post-SAH vasospastic ischemia—another possible etiology for our case—could not be definitively excluded but is less probable due to earlier than expected focal findings and brain CT lesion, absence of angiographic vasospasm in vessels irrigating the pontine basis in two angiographies together with the rarity of vasospasm in early nonaneurysmal PMSAH.

The syndrome of PMSAH together with PMI is a rarely reported but already well described pathological finding: Superficial perforator arterial rami rupture that explains all characteristics of the reported cases may cause some nonischemic PMSAH. We provisionally refer to these findings as PAramedian Pontine Perimesencephalic Hemorrhage Ischemic Syndrome (PAPPHIS) in order to call attention to other similar cases and confirm the existence of this construct that encompasses the main characteristics of our presentation in Table 1.

Ongoing observational study—PERFAN—led by Bern University and focused on aneurysmal perforator arteriopathy targeting 300 cases may bring important insights about the incidence of early concomitant brain ischemia in PMSAH cases. It will aid in estimating the frequency of cases with early ischemic-hemorrhage syndrome together with small vessel disease findings. Patients would benefit from early antiplatelet therapy, less invasive (and risky) investigations, faster etiology definition, mobilization, rehabilitation, and secondary prevention definition [10].

## Figures and Tables

**Table 1:** Alert criteria for paramedian pontine ischemia-perimesencephalic hemorrhage syndrome.

Main characteristics of PaPHIS presentation
Perimesencephalic SAH
Paramedian pontine infarction
Absence of vasospasm—artery dissection or mural hematoma
Absence of ruptured aneurysm, vascular malformation, or other bleeding source

Abbreviation: PaPPHIS = paramedian pontine perimesencephalic hemorrhage ischemic syndrome.

## Data Availability

The authors may share anonymously data about reported case under reasonable request.
